# *One Health Economics*: why and how economics should take on the interdisciplinary challenges of a promising public health paradigm

**DOI:** 10.3389/fpubh.2024.1379176

**Published:** 2024-05-30

**Authors:** Marc Leandri, Laurent Dalmas

**Affiliations:** UMI SOURCE, Université Paris-Saclay, UVSQ, IRD, Guyancourt, France

**Keywords:** one Health (OH) – approach, zoonose, antimicrobial resistance, economics, prevention

## Abstract

In this perspective paper, we argue that Economics could and should contribute to the development and implementation of the One Health approach currently emerging as a relevant interdisciplinary framework to address present and future infectious diseases. We show how proven tools from Health and Environmental Economics, such as burden evaluation, can be extended to fit the One Health multisectoral perspective. This global health framework could also benefit significantly from Economics to design efficient schemes for prevention and disease control. In return, adapting Economics to the challenges of One Health issues could pave the way for exciting developments in the Economics discipline itself, across many subfields.

## 1 Introduction: assessing the place of economics in the One Health paradigm

### 1.1 The One Health response to emerging health risks

Global change and increased flows of humans and goods across the planet are responsible for a significant rise of infectious diseases such as SARS, avian influenza and Ebola in the 21^st^ century (1). As the recent COVID-19 pandemic has shown, with over 1.5 million deaths and US$ trillions of losses worldwide in 2020 alone (2), these diseases exact a massive human and economic toll on our societies worldwide. As much as 60% of the current infectious diseases affecting humans (3) are zoonoses. As a result, human health and animal health are tightly intertwined when it comes to the origins, spread and burden of these pathologies. Through their multidimensional effects on humans, hosts and vectors, climate change and land-use change act as a catalyst on many of these zoonoses (4), thus reflecting the importance of environmental health on global health.

In response to these threats, the “One Health” paradigm has emerged as a cross-sectoral public health strategy to break down the barriers between human, animal and environmental health for both the analysis and the management of diseases. For Zinsstag et al. (5), “it promotes an integrated, systemic and unified approach of health at local, national and global levels, in order to better anticipate and tackle emerging diseases with pandemic risk, but also to adapt to present and future environmental impacts.” This strategy has been embraced by several major international institutions — the World Health Organization (WHO), the World Organization for Animal Health (OIE), the Food and Agriculture Organization of the United Nations (FAO) and the World Bank — as an efficient tool against future pandemics. According to the World Bank, a One Health approach could also generate substantial savings, as high as $6 billion per year, since “relatively modest investments in prevention will pay huge dividends” (6).

### 1.2 A limited explicit contribution of economics thus far

A handful of success stories (7) have demonstrated its potential, but the multi-scale implementation of this approach is still a work in progress and the WHO-OIE-FAO collaboration has not yet led to a concrete program (8). Beyond the difficulties entailed in establishing a true interdisciplinary collaboration between medicine and veterinary science, (9), there is a need to “encompass other disciplines that impact human health, such as economics, food security, and food safety” (10). Indeed, to our knowledge, the contribution of the social sciences and economics in particular to the conceptual and operational foundation of One Health has been limited so far. Our survey of the academic literature adopting the prism of economic analysis yielded three main works by non-economists and a World Bank report. Narrod et al. (11) propose a One Health framework for the economic evaluation of zoonoses that implies a modified risk analysis and insists on considering multi-sectoral impacts that go beyond simple control costs. Machalaba et al. (12) extend this initial take on impact valuation and recommend “system thinking” to help identify both the risks and the possibilities for their mitigation. Zinsstag et al. (13) show how financial issues can be integrated into One Health approaches, through four examples including brucellosis in Mongolia and the cost of bovine tuberculosis in Ethiopia. Although these case studies validate the recourse to economic valuation, they focus solely on monetary costs. Finally, the World Bank report (14) makes a well-documented economic case for the application of the One Health approach by assessing the net benefits of controlling zoonotic diseases.

This thin body of isolated contributions only skims the surface of the larger role Economics could play and shows the need to build progressively a consistent framework for One Health Economics. Hence our efforts in this paper to identify relevant lines of conjunction between the salient questions arising within the One Health approach and the conceptual tools Economics can provide. The science and policy-making of Public Health has long benefited from the contributions of Economics (15), whether it be through the direct mobilization of economic methods (e.g.: Cost–benefit Analysis, Discrete Choice Experiment) and indicators (e.g.: QALYs and DALYs) or through the parallel development of the subfield of Health Economics. Adapting economic constructs and instruments to the One Health research agenda thus appears to be an organic and legitimate step in pursuit of this collaboration.

## 2 A roadmap towards One Health Economics

Our analysis sketches out the premises of a non-exhaustive road map Economics could follow to support the growth of this integrated response to our century’s health and environmental challenges as well as its own development as a social science. Reflecting upon the potential input of Economics’ core principles, we find two relevant channels that can launch this convergence process before it widens further under the expected expansion of the One Health paradigm. First, the extensive theoretical and empirical techniques for monetary and non-monetary valuation that have been built by Economics, in particular in Health and Environmental Economics, must take on the integrated valuation of the new concept of impact that we define as the *“One Health” burden* and that is much broader than the standard “burden” of a disease often measured by Health economists. Second, keeping in mind that Economics is “the science which studies human behavior as a relationship between ends and scarce means which have alternative uses” (16), our discipline can help us understand the trade-offs underlying the individual decisions of adopting prevention measures against emerging diseases for human but also animal and environmental protection. As a result, One Health Economics can participate actively in the design and calibration of efficient instruments for prevention policies that are a major component of the One Health strategy (17). We shall structure each of these initial research avenues along two axes. On the one hand, we will distinguish the contribution of Economics in terms of analysis and policy-making. On the other hand, we will highlight the lowest-hanging economic fruits One Health can immediately grab before attempting a prospective assessment of the developments in Economics itself that will arise from this original outlook.

### 2.1 From compartmentalized health burdens to the *“One Health burden”*

One of the most visible contributions of Health Economics to Public Health policy-making is the valuation of the impacts of diseases. Economic indicators, including but not limited to monetary ones (15), can encompass mortality, morbidity, chronic loss of quality of life, as well as productivity losses and health care costs to quantify and synthesize the effects on humans, both individually and socially. Taking this contribution up to the One Health scale naturally implies encompassing the consequences for animal and environmental health as well. The first step in advancing from the concept of “economic burden” to a much broader “One Health burden” can be easily achieved by mobilizing separately the current economic valuation techniques developed by Health Economics for the impact on human capital, by Agricultural Economics for livestock capital and by Environmental Economics for natural capital. This additive approach is essential to allocate health care resources efficiently, but it remains compartmentalized. A more integrated second step toward constructing this “One Health burden” would require a targeted effort by Economics to recognize and value the feedback loops that take place between the three health dimensions at stake. For instance, Economics could engage, and even catalyze, an interdisciplinary dialog, so as to better understand the mechanisms of antimicrobial resistance across the human-animal-environmental nexus and subsequently assess its true cost from a holistic perspective. This goal should stimulate economic research on this issue, which has received little attention in our discipline so far (18, 19), especially from an integrated outlook. Because of the overuse of antimicrobials in animal (cattle breeding), environmental (agrochemicals) and human health, strains of resistant bacteria emerge and proliferate, generating massive losses in animal and human welfare and productivity (20). The global economic impacts of this new “tragedy of the commons” include a drop in international trade and livestock production and a lasting surge in health care costs. This could reduce annual gross domestic product by as much as 3.4% by 2050 (21), not mentioning the intangible costs of illnesses and deaths. Indeed, the global burden of antimicrobial resistance has been estimated around 5 million deaths/year for 2019 (22) and will undoubtedly rise if no concerted action is implemented. A cost–benefit analysis of the whole circuit of antimicrobial resistance could assess where and when vaccines and other alternatives to antibiotics for both humans and animals can prove much more cost-effective than excessive antimicrobial application.

The absolute valuation estimates of a “One Health burden” would significantly help policy makers assess the overall severity of a zoonotic disease and set its priority level on the public health agenda. As illustrated in Environmental Economics with the valuation of ecosystem services, the added value of economics transcends the numerical results produced: the valuation process itself fosters a pluridisciplinary effort to identify and link together the far-ranging impacts of a disease. In a pre-One Health contribution, Markandya et al. (23) demonstrated that substituting cow antibiotics by non-resistance inducing treatments in India was actually a much more cost-effective option than perceived, once the impact of this overuse on human (rabies and anthrax) and animal health (extinction of the vulture population) was taken into consideration in the economic calculus.

Beyond the burden itself, the economic valuation enterprise can also feed cost-efficiency analysis upscaled to the One Health transversal spheres. If a vaccination campaign can be deployed either among humans or among animals, or through a combination of both, as is already the case with influenza (24), economists can extend the standard one-dimensional frameworks of Pharmacoeconomics and Agricultural Economics to the two or three dimensions of the One Health approach.

### 2.2 Understanding health decisions across sectors and across countries

Another asset Economics should definitely bring to the One Health table is its capacity to study and model human behavior with policy design in mind. As shown by the COVID-19 pandemic, overcoming infectious diseases through prevention measures greatly rests on an understanding of the determinants of self-protection among a heterogeneous population. To that end, Public Health scientists have developed conceptual frameworks, such as the Theory of Planned Behavior and the Health Belief Model, which undergird a plethorical body of empirical studies on many health conditions and diseases. The fascinating similarities between these models and the strand of self-protection models in theoretical risk economics are just starting to be explored (25) and promise joint progress on efficient prevention policies. Economists should build on these initial interdisciplinary attempts in order to contribute to multidimensional prevention strategies, including preventive measures on humans and on livestock. Local empirical studies, embedded in a robust theoretical framework, could indeed better inform on the essential levers for human and animal prevention. The latter can depend on institutional environment and external financial constraints but also on intrinsic characteristics of the individual decision-maker, such as risk perception and risk aversion. As a result, prevention instruments such as information campaigns and nudges could be better targeted and efficiently implemented to achieve public health objectives while minimizing costs in the face of a changing environment. This shared goal could stimulate new research in Economics combining behavioral, risk and environmental issues to design innovative One Health policy mixes of subsidies, nudges, local and global information and education across sectors. The success of a One Health regulation strategy depends on the behavior of numerous agents across various sectors, requiring thorough analysis, aggregation, and guidance through public policies. For example, the challenge of antimicrobial resistance is traditionally addressed by Health Economics within the human sphere, employing game theoretical modeling. In a global economy, the overuse of antibiotics can be seen as a transboundary non-cooperative game among countries. Theoretical economic analysis can aid in designing a Pigouvian tax to internalize this decentralized market failure (26). The logical next step in the “One Health” approach would be to extend these models to include the strategic decisions of relevant agents affecting not only human but also animal and environmental antibiotic use, all subject to global market constraints.

Lastly, infusing economic analysis into One Health policy-making will automatically lead to differentiated One Health strategies depending on the local level of development. As soon as the economic trade-offs and financial stakes are properly accounted for, it becomes clear that the optimal One Health response to the same zoonotic disease could drastically differ between the North and the South. For instance, terminating preventively large numbers of livestock animals to regulate foot and mouth disease (13) and avian influenza and compensating their owners accordingly could prove an efficient strategy in the United Kingdom and France. But such a strategy would not be financially feasible in most low-to-medium-income countries, which have no choice but to focus on prevention in order to avoid dramatic income losses. Since some prevention measures, such as livestock vaccination, may also prove too costly for individual farmers, a comprehensive economic assessment of the One Health approach including both animal and human health benefits could justify international subsidies for efficient prevention impacting both local and global health. This demarcation line at the North–South frontier could structure the future field of One Health and will inevitably pose the question of international financing of global health.

A last dimension of individual behavior that One Health Economics can help address is the treatment applied to non-humans, such as slaughter campaigns to curb a zoonotic disease. The trade-offs between the efficiency of such a tool can be qualified by ethical considerations on animal welfare. Given the recent interest of Economics in animal welfare (27, 28), we can foresee in the medium-term ethical debates on the inclusion of animal welfare in the valuation of the “One Health burden” and on its technical implications if the intrinsic value of animals subject to a disease were to be considered alongside their instrumental value in the current anthropocentric One Health prism (29).

## 3 Conclusion

Two main axes organic to the core of Economics have emerged from our analysis of the potential for One Health Economics to contribute significantly to the public health policies of the future. These research avenues are far from capturing the comprehensive contributions of Economics, but we have shown that they can constitute solid building blocks for a broader research agenda benefiting both health policy-making and our discipline itself. The frontiers of One Health Economics will naturally extend from this initial structure to further questioning.

Beyond the immediate and operational public health objectives of One Health Economics, we believe they can contribute on a larger scale to the goal of transitioning toward a more sustainable society through its clear and vivid recognition of the fundamental interdependencies between the various spheres of life on Earth. As they are well-equipped to identify and express explicitly the benefits that humans can derive from this recognition (through monetary valuation for instance), economics could unlock the societal leverage of the “One Health” approach to make it a driving force behind a narrative on a transition that rethinks the interconnections between living beings. Within a broader perspective, the principle of articulating human, animal, and environmental health can indeed support a definition of sustainability as the systematic attention given to the interdependencies of living beings. And since this framework requires, by its very essence, to rely on interdisciplinary dialog, it can be thought as a system of knowledge, concepts, and rules to shape our *Anthropocene* era. One Health Economics could thus emerge as a key field within the *episteme* (30) of our epoch that will lead to sustainable policies through interdisciplinary research.

## Data availability statement

The original contributions presented in the study are included in the article/Supplementary material, further inquiries can be directed to the corresponding author/s.

## Author contributions

ML: Conceptualization, Investigation, Writing – original draft, Writing – review & editing. LD: Conceptualization, Investigation, Writing – original draft, Writing – review & editing.
