# Supplementary material for: One Health Economics: why and how economics should take on the interdisciplinary challenges of a promising public health paradigm
Source: Front Public Health. 2024 May 30;12:1379176. doi: 10.3389/fpubh.2024.1379176 (PMC11177617; doi:10.3389/fpubh.2024.1379176)
Supplement: Supplementary file 1 [file Data_Sheet_1.docx]

***One Health Economics*: why and how Economics should take on the interdisciplinary challenges of a promising public health paradigm.**

**Abstract:** In this perspective paper we argue that Economics could and should contribute to the development and implementation of the One Health approach currently emerging as a relevant interdisciplinary framework to address present and future infectious diseases. We show how proven tools from Health and Environmental Economics, such as burden evaluation, can be extended to fit the One Health multisectoral perspective. This global health framework could also benefit significantly from Economics to design efficient schemes for prevention and disease control. In return, adapting Economics to the challenges of One Health issues could pave the way for exciting developments in the Economics discipline itself, across many subfields.

1. **Introduction: assessing the place of Economics in the One Health Paradigm**
   1. **The One Health response to emerging health risks**

Global change and increased flows of humans and goods across the planet are responsible for a significant rise of infectious diseases such as SARS, avian influenza and Ebola in the 21^st^ century (Baker et al., 2022). As the recent COVID-19 pandemic has shown, with over 1.5 million deaths and US$ trillions of losses worldwide in 2020 alone (Kaplan et al., 2022), these diseases exact a massive human and economic toll on our societies worldwide. As much as 60% of the current infectious diseases affecting humans (Jones et al., 2008) are zoonoses. As a result, human health and animal health are tightly intertwined when it comes to the origins, spread and burden of these pathologies. Through their multidimensional effects on humans, hosts and vectors, climate change and land-use change act as a catalyst on many of these zoonoses (Rupasinghe et al., 2022), thus reflecting the importance of environmental health on global health.

In response to these threats, the “One Health” paradigm has emerged as a cross-sectoral public health strategy to break down the barriers between human, animal and environmental health for both the analysis and the management of diseases. For Zinsstag et al. (2022b), “it promotes an integrated, systemic and unified approach of health at local, national and global levels, in order to better anticipate and tackle emerging diseases with pandemic risk, but also to adapt to present and future environmental impacts”. This strategy has been embraced by several major international institutions — the World Health Organization (WHO), the World Organization for Animal Health (OIE), the Food and Agriculture Organization of the United Nations (FAO) and the World Bank — as an efficient tool against future pandemics. According to the World Bank, a One Health approach could also generate substantial savings, as high as $6 billion per year, since “relatively modest investments in prevention will pay huge dividends” (World Bank, 2022).

- 1. **A limited explicit contribution of Economics thus far**

A handful of success stories (Gibson et al., 2022) have demonstrated its potential, but the multi-scale implementation of this approach is still a work in progress and the WHO-OIE-FAO collaboration has not yet led to a concrete program (Giraudoux et al., 2022). Beyond the difficulties entailed in establishing a true interdisciplinary collaboration between medicine and veterinary science, (Ribeiro et al., 2019), there is a need to “encompass other disciplines that impact human health, such as economics, food security, and food safety” (World Health Organization, 2012). Indeed, to our knowledge, the contribution of the social sciences and economics in particular to the conceptual and operational foundation of One Health has been limited so far. Our survey of the academic literature adopting the prism of economic analysis yielded three main works by non-economists and a World Bank report. Narrod et al. (2012) propose a One Health framework for the economic evaluation of zoonoses that implies a modified risk analysis and insists on considering multi-sectoral impacts that go beyond simple control costs. Machalaba et al. (2017) extend this initial take on impact valuation and recommend “system thinking” to help identify both the risks and the possibilities for their mitigation. Zinsstag et al. (2022a) show how financial issues can be integrated into One Health approaches, through four examples including brucellosis in Mongolia and the cost of bovine tuberculosis in Ethiopia. Although these case studies validate the recourse to economic valuation, they focus solely on monetary costs. Finally, the World Bank report (World Bank, 2012) makes a well-documented economic case for the application of the One Health approach by assessing the net benefits of controlling zoonotic diseases.

This thin body of isolated contributions only skims the surface of the larger role Economics could play and shows the need to build progressively a consistent framework for One Health Economics. Hence our efforts in this paper to identify relevant lines of conjunction between the salient questions arising within the One Health approach and the conceptual tools Economics can provide. The science and policy-making of Public Health has long benefited from the contributions of Economics (Edwards et al., 2013), whether it be through the direct mobilization of economic methods (e.g.: Cost-benefit Analysis, Discrete Choice Experiment) and indicators (e.g.: QALYs and DALYs) or through the parallel development of the subfield of Health Economics. Adapting economic constructs and instruments to the One Health research agenda thus appears to be an organic and legitimate step in pursuit of this collaboration.

1. **A roadmap towards One Health Economics**

Our analysis sketches out the premises of a non-exhaustive road map Economics could follow to support the growth of this integrated response to our century’s health and environmental challenges as well as its own development as a social science. Reflecting upon the potential input of Economics’ core principles, we find two relevant channels that can launch this convergence process before it widens further under the expected expansion of the One Health paradigm. First, the extensive theoretical and empirical techniques for monetary and non-monetary valuation that have been built by Economics, in particular in Health and Environmental Economics, must take on the integrated valuation of the new concept of impact that we define as the *“One Health” burden* and that is much broader than the standard “burden” of a disease often measured by Health economists. Second, keeping in mind that Economics is “the science which studies human behavior as a relationship between ends and scarce means which have alternative uses” (Robbins, 1935), our discipline can help us understand the trade-offs underlying the individual decisions of adopting prevention measures against emerging diseases for human but also animal and environmental protection. As a result, One Health Economics can participate actively in the design and calibration of efficient instruments for prevention policies that are a major component of the One Health Strategy (Kelly et al., 2020). We shall structure each of these initial research avenues along two axes. On the one hand, we will distinguish the contribution of Economics in terms of analysis and policy-making. On the other hand, we will highlight the lowest-hanging economic fruits One Health can immediately grab before attempting a prospective assessment of the developments in Economics itself that will arise from this original outlook.

- 1. **From compartmentalized health burdens to the *“One Health burden”***

One of the most visible contributions of Health Economics to Public Health policy-making is the valuation of the impacts of diseases. Economic indicators, including but not limited to monetary ones (Edwards et al., 2013), can encompass mortality, morbidity, chronic loss of quality of life, as well as productivity losses and health care costs to quantify and synthesize the effects on humans, both individually and socially. Taking this contribution up to the One Health scale naturally implies encompassing the consequences for animal and environmental health as well. The first step in advancing from the concept of “economic burden” to a much broader “One Health burden” can be easily achieved by mobilizing separately the current economic valuation techniques developed by Health Economics for the impact on human capital, by Agricultural Economics for livestock capital and by Environmental Economics for natural capital. This additive approach is essential to allocate health care resources efficiently, but it remains compartmentalized. A more integrated second step towards the construct of this “One Health burden” would require a targeted effort by Economics to recognize and value the feedback loops that take place between the three health dimensions at stake. For instance, Economics could engage, and even catalyze, an interdisciplinary dialogue, so as to better understand the mechanisms of antimicrobial resistance across the human-animal-environmental nexus and subsequently assess its true cost from a holistic perspective. This goal should stimulate economic research on this issue, which has received little attention in our discipline so far (Laxminarayan and Brown, 2001; Roope et al., 2019), especially from an integrated outlook. Because of the overuse of antimicrobials in animal (cattle breeding), environmental (agrochemicals) and human health, strains of resistant bacteria emerge and proliferate, generating massive losses in animal and human welfare and productivity (Velazquez-Meza et al., 2022). A cost-benefit analysis of the whole circuit of antimicrobial resistance could assess where and when vaccines and other alternatives to antibiotics for both humans and animals can prove much more cost-effective than excessive antimicrobial application.

The absolute valuation estimates of a “One Health burden” would significantly help policy makers assess the overall severity of a zoonotic disease and set its priority level on the public health agenda. As illustrated in Environmental Economics with the valuation of ecosystem services, the added value of economics transcends the numerical results produced: the valuation process itself fosters a pluridisciplinary effort to identify and link together the far-ranging impacts of a disease. Beyond the burden itself, the economic valuation enterprise can also feed cost-efficiency analysis upscaled to the One Health transversal spheres. If a vaccination campaign can be deployed either among humans or among animals, or through a combination of both, as is already the case with influenza (Carpenter et al., 2022), economists can extend the standard one-dimensional frameworks of Pharmacoeconomics and Agricultural Economics to the two or three dimensions of the One Health approach.

- 1. **Understanding health decisions across sectors and across countries**

Another asset Economics should definitely bring to the One Health table is its capacity to study and model human behavior with policy design in mind. As shown by the COVID-19 pandemic, overcoming infectious diseases through prevention measures greatly rests on an understanding of the determinants of self-protection among a heterogeneous population. To that end, Public Health scientists have developed conceptual frameworks, such as the Theory of Planned Behavior and the Health Belief Model, which undergird a plethorical body of empirical studies on many health conditions and diseases. The fascinating similarities between these models and the strand of self-protection models in theoretical risk economics are just starting to be explored (Augeraud and Leandri, 2023) and promise joint progress on efficient prevention policies. Economists should build on these initial interdisciplinary attempts in order to contribute to multidimensional prevention strategies, including preventive measures on humans and on livestock. Local empirical studies, embedded in a robust theoretical framework, could indeed better inform on the essential levers for human and animal prevention. The latter can depend on institutional environment and external financial constraints but also on intrinsic characteristics of the individual decision-maker, such as risk perception and risk aversion. As a result, prevention instruments such as information campaigns and nudges could be better targeted and efficiently implemented to achieve public health objectives while minimizing costs in the face of a changing environment. This shared goal could stimulate new research in Economics combining behavioral, risk and environmental issues to design innovative One Health policy mixes of subsidies, nudges, local and global information and education across sectors. Lastly, infusing economic analysis into One Health policy-making will automatically lead to differentiated One Health strategies depending on the local level of development. As soon as the economic trade-offs and financial stakes are properly accounted for, it becomes clear that the optimal One Health response to the same zoonotic disease could drastically differ between the North and the South. For instance, terminating preventively large numbers of livestock animals to regulate foot and mouth disease (Zinstag et al., 2015) and avian influenza and compensating their owners accordingly could prove an efficient strategy in the United Kingdom and France. But such a strategy would not be financially feasible in most low-to-medium-income countries, which have no choice but to focus on prevention in order to avoid dramatic income losses. Since some prevention measures, such as livestock vaccination, may also prove too costly for individual farmers, a comprehensive economic assessment of the One Health approach including both animal and human health benefits could justify international subsidies for efficient prevention impacting both local and global health.

**Conclusion**

Two main axes organic to the core of Economics have emerged from our analysis of the potential for One Health Economics to contribute significantly to the public health policies of the future. These research avenues are far from capturing the comprehensive contributions of Economics, but we have shown that they can constitute solid building blocks for a broader research agenda benefiting both health policy-making and our discipline itself. The frontiers of One Health Economics will naturally extend from this initial structure to further questioning. For instance, given the recent interest of Economics in animal welfare (Treich, 2022; Budolfson et al., 2023), we can foresee in the medium-term ethical debates on the inclusion of animal welfare in the valuation of the “One Health burden” and on its technical implications if the intrinsic value of animals subject to a disease were to be considered alongside their instrumental value in the current anthropocentric One Health prism (Coghlan et al., 2021). Another demarcation line that could structure the field of One Health Economics should be explored at the North-South frontier and will inevitably pose the question of international financing of global health.

Words: 2935

**REFERENCES**

## Augeraud-Véron, E. and Leandri, M. Optimal self-protection and health risk perception: bridging the gap between risk theory and the Health Belief Model, *EconomiX Working Paper*. **12** (2023).

Baker, R. E., Mahmud, A. S., Miller, I. F., Rajeev, M., Rasambainarivo, F., Rice, B. L., ... & Metcalf, C. J. E.. Infectious disease in an era of global change. *Nature Reviews Microbiology*. **20(4)**, 193-205 (2022).

Budolfson, M., Fischer, B., & Scovronick, N. Animal welfare: Methods to improve policy and practice. *Science*. **381(6653),** 32-34 (2023).

Carpenter, A., Waltenburg, M. A., Hall, A., Kile, J., Killerby, M., Knust, B., ... & Vaccine Preventable Zoonotic Disease Working Group. Vaccine preventable zoonotic diseases: challenges and opportunities for public health progress. *Vaccines*. **10(7)**, 993 (2022).

Coghlan, S., Coghlan, B. J., Capon, A., & Singer, P. (2021). A bolder One Health: expanding the moral circle to optimize health for all. *One Health Outlook*, 3, 1-4.

Edwards, R. T., Charles, J. M., & Lloyd-Williams, H. (2013). Public health economics: a systematic review of guidance for the economic evaluation of public health interventions and discussion of key methodological issues. *BMC public health*, 13, 1-13.

Gibson, A. D., Yale, G., Corfmat, J., Appupillai, M., Gigante, C. M., Lopes, M., ... & Mellanby, R. J. Elimination of human rabies in Goa, India through an integrated One Health approach. *Nature Communications*. **13(1)**, 2788 (2022).

Jones, K. E., Patel, N. G., Levy, M. A., Storeygard, A., Balk, D., Gittleman, J. L., & Daszak, P. Global trends in emerging infectious diseases. *Nature*. **451(7181)**, 990-993 (2008).

Kaplan, S., Lefler, J., & Zilberman, D. The political economy of COVID‐19. *Applied Economic Perspectives and Policy*. **44(1)**, 477-488 (2022).

Kelly, Terra R., Catherine Machalaba, William B. Karesh, Paulina Zielinska Crook, Kirsten Gilardi, Julius Nziza, Marcela M. Uhart, et al. Implementing One Health approaches to confront emerging and re-emerging zoonotic disease threats: lessons from PREDICT. *One Health Outlook*. **2(1)**, 1 (2020).

Laxminarayan, R., & Brown, G. M. Economics of antibiotic resistance: a theory of optimal use. *Journal of Environmental Economics and* Management. **42(2)**, 183-206 (2001).

Machalaba, Catherine, Kristine M Smith, Lina Awada, Kevin Berry, Franck Berthe, Timothy A Bouley, Mieghan Bruce, et al. One Health Economics to confront disease threats. *Transactions of The Royal Society of Tropical Medicine and Hygiene*, **111(6)**, 235‑37 (2017).

Giraudoux, P., Besombes, C., Bompangue, D., Guégan, J. F., Mauny, F., & Morand, S. One Health or ‘One Health washing’? An alternative to overcome now more than ever. *CABI One Health* (2022).

Narrod, Clare, Jakob Zinsstag, et Marites Tiongco. A One Health Framework for Estimating the Economic Costs of Zoonotic Diseases on Society. *EcoHealth*, **9(2)**, 150‑62 (2012).

Ribeiro, C. D. S., van de Burgwal, L. H., & Regeer, B. J. Overcoming challenges for designing and implementing the One Health approach: A systematic review of the literature. *One Health*, **7**, 100085 (2019).

Roope, L. S., Smith, R. D., Pouwels, K. B., Buchanan, J., Abel, L., Eibich, P., ... & Wordsworth, S. The challenge of antimicrobial resistance: what economics can contribute. *Science*, **364(6435)**, 4679 (2019).

Rupasinghe, R., Chomel, B. B., & Martínez-López, B. Climate change and zoonoses: A review of the current status, knowledge gaps, and future trends. *Acta Tropica*, **226**, 106225 (2022).

Treich, N. The Dasgupta Review and the problem of anthropocentrism. *Environmental and Resource Economics*, **83(4)**, 973-997 (2022).

Velazquez-Meza, M. E., Galarde-López, M., Carrillo-Quiróz, B., & Alpuche-Aranda, C. M. Antimicrobial resistance: one health approach. *Veterinary World*, **15(3)**, 743 (2022).

World, Bank. People, Pathogens And Our Planet - Volume 2, *The Economics of One Health*, Washington, DC (2012).

World Bank. Putting Pandemics Behind Us: Investing in One Health to Reduce Risks of Emerging Infectious Diseases. Washington, DC (2022).

Zinsstag, J., A. Choudhury, F. Roth, et A. Shaw. One Health Economics. In Zinsstag et al. (Eds), *One Health: The Theory and Practice of Integrated Health Approaches*, CABI, 134‑45 (2022a).

Zinsstag, J., Schelling, E., Crump, L., Whittaker, M., Tanner, M., & Stephen, C. (Eds.). *One Health: the theory and practice of integrated health approaches*. CABI (2022b).
